# Discovery of the genus *Glyphicnemis* Förster in the Oriental Region (Hymenoptera, Ichneumonidae, Cryptinae)

**DOI:** 10.3897/zookeys.678.12397

**Published:** 2017-06-07

**Authors:** Tao Li, Mao-Ling Sheng, Kyohei Watanabe, Zheng-Fu Guo

**Affiliations:** 1 General Station of Forest Pest Management, State Forestry Administration, Shenyang 110034, P. R. China; 2 Kanagawa Prefectural Museum of Natural History, Iriuda 499, Odawara, Kanagawa 250–0031, Japan; 3 Jiangxi Forestry Society, Nanchang Jiangxi 330038, P.R. China

**Keywords:** *Glyphicnemis*, key, new species, Phygadeuontini, taxonomy

## Abstract

The genus *Glyphicnemis* Förster, 1869 is newly recorded from the Oriental Region based on a new species, *Glyphicnemis ganica* Sheng & Li, **sp. n.**, collected from Jiangxi Province, in the oriental part of China. This species resembles *G. watanabei* (Uchida, 1930) from Japan in the coloration of flagellum and the structure and colouration of metasomal tergites, but it can be distinguished by the density and length of clypeal setae, the large propodeal spiracle, and the sculpture of area superomedia. Illustrations of *G. ganica* and *G. watanabei* are provided. A key to the Asian species of this genus is also given.

## Introduction

*Glyphicnemis* Förster, 1869, belonging to the tribe Phygadeuontini of the subfamily Cryptinae (Hymenoptera: Ichneumonidae), comprises 12 species (Yu et al. 2016), of which six are from the Eastern Palaearctic Region (Ghahari and Jussila 2014, Jonaitis 1981, Uchida 1930, 1952) (three of them are found across the Palaearctic), five from the Western Palaearctic Region (Ciochia 1973, Jonaitis 1981, Sawoniewicz 1985, Schwarz and Shaw 2010, Yu et al. 2016) and four from the Nearctic Region (Luhman 1986). One species of *Glyphicnemis* Förster was known from China (Uchida 1930). The diagnostic characters of the genus were most recently revised by Townes (1970).

Jonaitis (1981) provided a key to the species of the European part of USSR. Sawoniewicz (1985) revised the European species of the subtribe Endaseina with keys to the genera and the species of *Glyphicnemis*. Most Eastern Palaearctic species were described by Uchida (1930, 1952, 1955). A single species, *G. satoi* (Uchida, 1930), is previously recorded from Heilongjiang Province, Northeastern China.

In this article a new species of *Glyphicnemis* from Jiangxi, China, is described. This species is the first record of this genus from the Oriental Region.

## Materials and methods

Specimens were collected with interception traps (IT) (Li et al. 2012) in Wugongshan National Natural Reserve, Pingxiang, Jiangxi Province, P.R. China. Type specimens are deposited in the Insect Museum, General Station of Forest Pest Management (GSFPM), State Forestry Administration, People’s Republic of China.

The type specimens of *Stylocryptus osakensis* Uchida, 1930 (Holotype), *S. satoi* Uchida, 1930 (Lectotype), *S. watanabei* Uchida, 1930 (Lectotype), deposited in Hokkaido University, Japan, were examined and compared to the new species. *Glyphicnemis atrata* (Strobl, 1901), *G. vagabunda* (Gravenhorst, 1829) and *G. profligator* (Fabricius, 1775), deposited in Zoologische Staatssammlung München, München, Germany and identified by Sawoniewicz, were also compared to the new species.

Images were taken using a Stereomicroscope (Leica M205A) with a LAS Montage MultiFocus. Morphological terminology is mostly based on Gauld (1991).

## Taxonomy

### Glyphicnemis

Taxon classificationAnimaliaHymenopteraIchneumonidae

Förster, 1869

Glyphicnemis Förster, 1869: 181, figs 2,3,6,9,10.

#### Diagnosis.

Eye surface usually with sparse, short hairs (Fig. 3). Clypeus very wide, apical margin thick. Lower tooth of mandible much longer than upper tooth (Fig. 2). Upper end of epicnemial carina reaching to subalar prominence. Scutoscutellar groove with strong median longitudinal carina (Fig. 6). Outer side and apex of tibiae with strong spines (Figs 9, 10). Apical truncation of hind tibia very oblique (Fig. 9). Spurs of hind tibia inserted distinctly before apex (Fig. 10). Median dorsal carina of first tergite strong.

#### Key to species of *Glyphicnemis* recorded from Asia (Oriental and Eastern Palaearctic Regions) (Female only)

**Table:** 

1	Propodeal spiracle small, semicircular, 1.3–1.5 × as long as wide. Costula connecting area superomedia approximately at its middle. Tergites 2 and 3 usually reddish brown.	***G. atrata* (Strobl)**
–	Propodeal spiracle large, elongate, 1.5–2.1 × as long as wide. Costula connecting area superomedia at its posterior portion (in *G. profligator* and *G. vagabunda* at midlength), or tergites 2 and 3 black	**2**
2	Flagellum red, apical portion brown-black, without white ring. Tergites (except base of first tergite which is black) and hind femur red-brown	***G. vagabunda* (Gravenhorst)**
–	Flagellum with white ring (Fig. 4), at least dorsal median portion white. Anterior and posterior extremities of tergites black or brownish black (except *G. profligator*). Hind femur black, brown-black, red-brown, or light colored	**3**
3	Hypostomal carina distinctly higher than genal carina. Area superomedia distinctly wider than its length	***G. profligator* (Fabricius)**
–	Hypostomal carina almost as high as genal carina. Area superomedia almost as wide as long	**4**
4	Tergites 2 and 3 smooth, shiny, without punctures, or almost impunctate; black or brownish black	**5**
–	Tergites 2 and 3 more or less granulate, with fine punctures; red or darkish red-brown	**6**
5	Apical margin of clypeus with dense long hairs (Fig. 12). Propodeal spiracle large, elliptic. Area superomedia smooth (Fig. 13). Tergites darkish red-black. Median portion of hind tibia yellow	***G. watanabei* (Uchida)**
–	Apical margin of clypeus without exceptional long hairs (Fig. 2). Propodeal spiracle elongate, 2 × as long as wide (Figs 7, 8). Area superomedia (Fig. 11) with dense irregular transverse rugae. Second and subsequent tergites black (Fig. 1). Dorsal side of hind tibia darkish brown, ventral side yellow-brown (Figs 1, 9)	***G. ganica* Sheng & Li, sp.n.**
6	Anterior portion of postpetiole with transverse rugae, posterior portion with longitudinal rugae. Lateral carinae of area superomedia very weak, costula connecting approximately at its posterior 0.25. Tergites 2 and 3 darkish red-brown	***G. satoi* (Uchida)**
–	Median portion of postpetiole smooth, almost unpunctate, lateral portion with sparse fine punctures. Lateral carinae of area superomedia strong, costula connecting almost at its middle. Tergites 2 and 3 red	***G. osakensis* (Uchida)**

### Glyphicnemis ganica

Taxon classificationAnimaliaHymenopteraIchneumonidae

Sheng & Li sp. n.

http://zoobank.org/EEA5AC71-4536-40C1-961B-8D00C25F0124

Figs 1–3 , 4–9 , 10–11

#### Type material.

Holotype, female, Hongyangu, Wugongshan Natural Reserve, 530m, 24 May 2016, collected with IT by Yu Yao (GSFPM).

#### Diagnosis.

**Figures 1–3.**
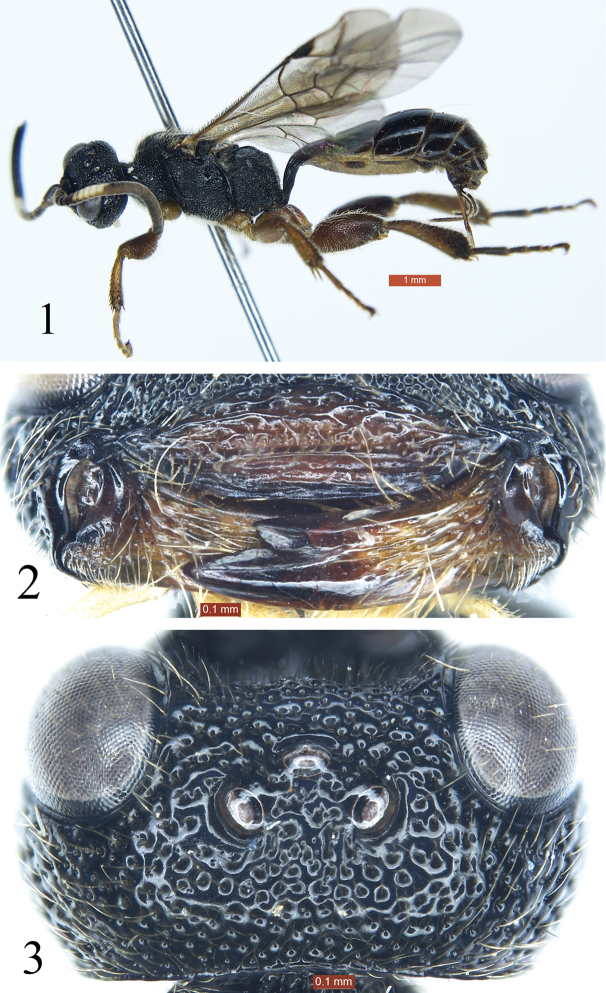
*Glyphicnemis ganica* sp. n. Holotype. Female. **1** Habitus, lateral view **2** Clypeus and mandibles **3** Head, dorsal view.

Subapical portion of clypeus strongly convex, forming a transverse ridge, apical margin without exceptional long hairs (Fig. 2). Area superomedia with dense, irregular transverse rugae (Fig. 11). Propodeal spiracle large, elongate, approximately 2 × as long as wide (Figs 7, 8). Second and subsequent tergites black (Fig. 1). Dorsal side of hind tibia darkish brown, ventral side yellow-brown (Fig. 9). First tergite dark brown, posterior portion of postpetiole red-brown. Second and subsequent tergites black.

#### Description.

**Female**. Body length approximately 8.5 mm. Forewing length approximately 6.0 mm. Ovipositor sheath length 1.2 mm. Head, mesosoma, and apical portion of metasoma with dense short yellowish brown hairs.

**Figures 4–9.**
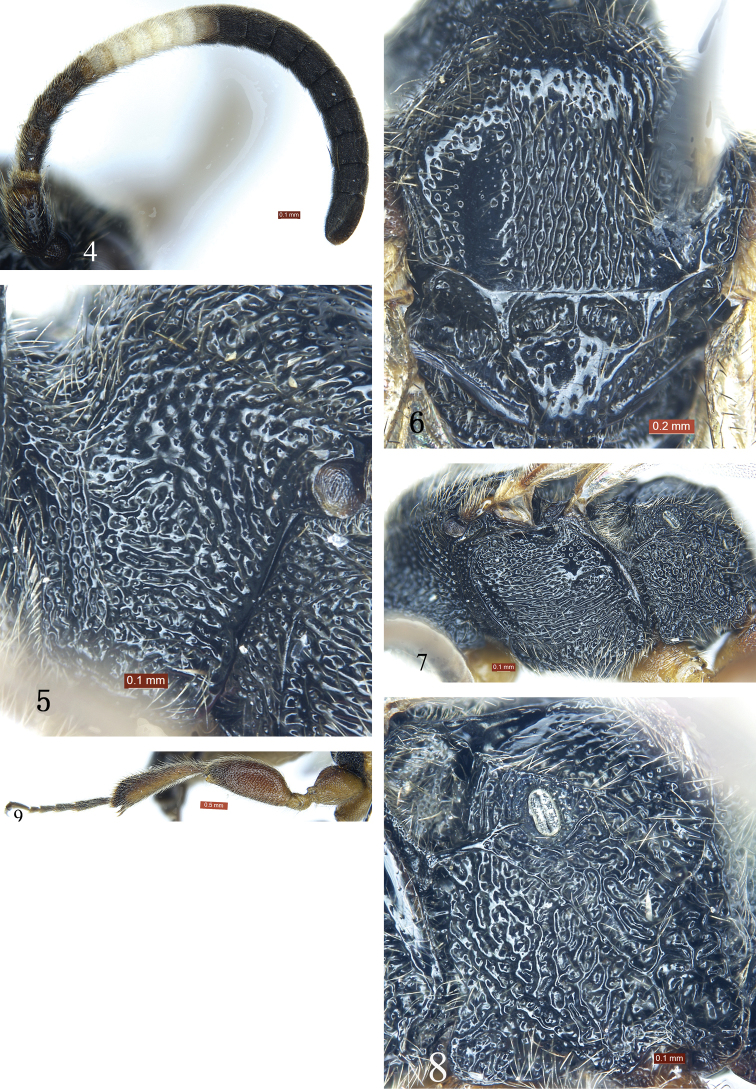
*Glyphicnemis ganica* sp. n. Holotype. Female. **4** Antenna **5** Pronotum, lateral view **6** Mesoscutum and scutellum **7** Mesosoma, lateral view **8** Metapleuron **9** Hind leg.

*Head*. With dense large punctures. Face 2.8 × as wide as long, strongly convex. Clypeus 4.0 × as wide as long (Fig. 2); basal portion with transverse rugae; subapical portion strongly convex, forming a transverse ridge. Basal portion of mandibles with longitudinal rugae and fine punctures; lower tooth 3.7 × as long as upper tooth. Eye particularly small, with sparse short hairs. Malar space 0.4 × as long as basal width of mandible. Gena in lateral view 1.4 × as long as width of eye, with punctures larger than those of face. Vertex (Fig. 3) with dense uneven puctures. Postocellar line 1.2 × as long as ocular-ocellar line. Antenna (Fig. 4) short, with 19 flagellomeres. Second flagellomere 1.25 × as long as maximum width. Ratio of length from first to fifth flagellomeres: 1.4:1.0:0.9:0.8:0.7. Occipital carina complete.

**Figures 10–11.**
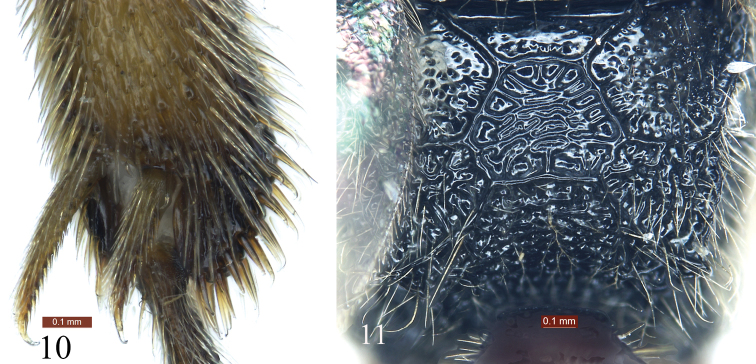
*Glyphicnemis ganica* sp. n. Holotype. Female. **10** Apical portion of hind tibia **11** Propodeum.

*Mesosoma*. Anterior portion of pronotum laterally (Fig. 5) with dense irregular rugae and punctures; lateral concavity with uneven transverse rugae; upper posterior portion with large punctures. Epomia distinct. Mesoscutum (Fig. 6) shiny, with irregular punctures, postero-median portion with longitudinal rugae. Scutellum (Fig. 6) slightly convex, smooth, shiny, with sparse punctures. Upper portion of mesopleuron (Fig. 7) with dense, irregular punctures, lower portion with transverse rugae and irregular, indistinct punctures; lower posterior portion with oblique rugae. Speculum small, smooth, shiny. Metapleuron (Fig. 8) with strong, irregular reticulate rugae. Wings gray, hyaline. Fore wing with vein 1*cu*-*a* distinctly distal of 1-*M.* Areolet pentagonal. Distance from vein 2*rs*-*m* to 2*m*-*cu* slightly longer than distance from 2*m*-*cu* to 3*rs*-*m.* Vein 2-*Cu* approximately 2 × as long as 2*cu*-*a.* Hind wing vein 1-*cu* about 3 × as long as *cu*-*a*; 1-*cu* strongly inclivous. Hind leg (Figs 9, 10) exceptionally stout. Hind femur 2.5 × as long as its maximum width. Ratio of length of one to fifth hind tarsomeres 2.0:1.0:0.7:0.4:1.0. Propodeum (Fig. 11) with complete carinae. Area basalis shiny, with sparse fine punctures, strongly convergent posteriorly. Area superomedia hexagonal, with strong irregular transverse rugae, costula connecting approximately at its posterior 0.3. Area petiolaris strongly slant, with irregular transverse rugae. Area externa shiny, with distinct, uneven punctures. Area dentipara and area lateralis with irregular reticulate rugae. Propodeal spiracle (Fig. 8) elongate, approximately twice as long as wide.

*Metasoma*. Tergites smooth, shiny. First tergite 1.7 × as long as posterior width, median dorsal carinae reaching about 0.6 of first tergite; posterolateral parts with sparse fine punctures. Dorsolateral and ventrolateral carinae complete. Spiracle circular, small, located at posterior 0.3 of first tergite. Second tergite 0.56 × as long as its posterior width, with a few indistinct fine punctures. Third tergite 0.7 × as long as its posterior width, 0.8 × as long as its posterior width. Fourth and subsequent tergites with short brown pubescence. Ovipositor sheath 0.9 × as long as hind tibia.

*Coloration* (Fig. 1). Black, except for the following. Clypeus, mandibles except teeth, reddish brown. Maxillary and labial palpi fawn. Ventral side of scape and pedicel reddish brown. Ventral side of flagellum slightly brownish. Flagellomeres 5 to 9 white, ventral side narrowly slightly blackish. Dorsal sides of legs red-brown, ventral sides yellow-brown; apical portion of hind tibia and tarsi more or less brownish black. Tegulae and posterior portion of postpetiole red-brown. First tergite dark brown. Pterostigma and veins brownish black.

#### Remarks.

**Figures 12–13.**
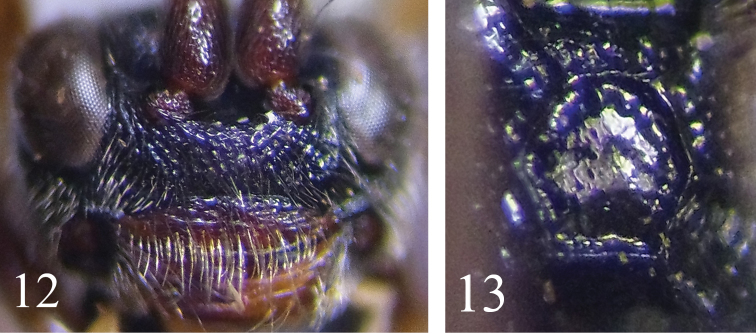
*Glyphicnemis watanabei* (Uchida, 1930). Holotype. Female. **12** Head, anterior view **13** Propodeum.

This new species is similar to *G. watanabei* (Uchida, 1930) but can be distinguished from the latter by the following combination of characters: apical margin of clypeus without unusual long hairs (vs. with dense, long hairs; see Fig. 12). Area superomedia with strong irregular transverse rugae, costula connecting at its posterior 0.3 (vs. smooth, shiny, without rugae, costula connecting slightly beyond its middle; see Fig. 13). Ovipositor sheath 0.9 × as long as hind tibia (vs. 0.75). Clypeus entirely reddish brown (vs. basally black, apically brown). First tergite dark brown (vs. black). All coxae and hind femur yellow brown (vs. black). It can also be distinguished from the known species of the Oriental and Eastern Palaearctic Regions by the preceding key.

#### Etymology.

The specific name is derived from the type locality.

## Supplementary Material

XML Treatment for Glyphicnemis

XML Treatment for Glyphicnemis ganica
